# Inter-Specimen Imbalance of Mitochondrial Gene Copy Numbers Predicts Clustering of *Pneumocystis jirovecii* Isolates in Distinct Subgroups

**DOI:** 10.3390/jof4030084

**Published:** 2018-07-10

**Authors:** Cara Mia Dunaiski, Lena Janssen, Hannah Erzinger, Monika Pieper, Sarah Damaschek, Oliver Schildgen, Verena Schildgen

**Affiliations:** Kliniken der Stadt Köln gGmbH, Institut für Pathologie, Klinikum der Privaten Universität Witten/Herdecke, 51109 Köln, Germany; duncarmia@gmail.com (C.M.D.); lena.janssen@uni-wh.de (L.J.); hannah.erzinger@uni-wh.de (H.E.); pieperm@kliniken-koeln.de (M.P.); damascheks@kliniken-koeln.de (S.D.); schildgeno@kliniken-koeln.de (O.S.)

**Keywords:** PCP, quantification, *Pneumocystis jirovecii*

## Abstract

The molecular detection of *Pneumocystis jirovecii* is an important therapy-relevant tool in microbiological diagnostics. However, the quantification of this pathogen in the past has revealed discordant results depending on the target gene. As the clinical variety of *P. jirovecii* infections ranges between life-threatening infections and symptom-free colonization, the question arises if qPCRs are reliable tools for quantitative diagnostics of *P. jirovecii*. *P. jirovecii* positive BALs were quantitatively tested for the copy numbers of one mitochondrial (COX-1) and two nuclear single-copy genes (KEX1 and DHPS) compared to the mitochondrial large subunit (mtLSU) by qPCR. Independent of the overall mtLSU copy number *P. jirovecii* clustered into distinct groups based on the ratio patterns of the respective qPCRs. This study, which compared different mitochondrial to nuclear gene ratio patterns of independent patients, shows that the mtLSU gene represents a highly sensitive qPCR tool for the detection of *P. jirovecii*, but does not display a reliable target for absolute quantification.

## 1. Introduction

*Pneumocystis* has a long history, spanning its discovery in 1909 as a form of *Trypanosoma cruzi* [1], its detection in rats and its classification as a new parasitic species in 1912 [2] and its relationship with pneumonia in the 1950s [3]. Its clinical relevance became evident with the onset of the global AIDS epidemic in the 1980s, which classified *Pneumocystis* as an AIDS-defining illness [4]. Initially, the organism was classified as a protozoan, but the upcoming gene sequencing approaches in the late 1980s provided the taxonomic evidence that *Pneumocystis carinii* belongs to the kingdom of fungi [5,6,7]. Before the classification of *P. jirovecii* in 2001 as the human species based on 18s rRNA sequence variation all *Pneumocystis* strains were identified as *P. carinii* [8].

*P. jirovecii* is an opportunistic, respiratory, ascomycetous fungus that causes numerous pulmonary infections in humans. It is assumed that healthy individuals are asymptomatic carriers of the fungus and clinical presentation usually appears in an immunocompromised state. Thereby, *Pneumocystis* pneumonia (PCP) is the most severe manifestation of *P. jirovecii* infection and is an important cause of morbidity and mortality in AIDS and other immunocompromised patients.

Thereby, due to the compartmentalization of lungs with different genotypes [9] a change of genotypes in recurrent PCP episodes could be either by serial infections or by (re-)activation of a genotype being present in a different lung compartment. *Pneumocystis* is not only an important pathogen, especially in non-industrialized countries, but is the second-most life threatening, invasive fungal infection worldwide, with an estimated number of 400,000 cases per year [10]. Even though mortality to PCP in HIV patients has declined over the years, the mortality linked to PCP in non-AIDS patients is relatively high [11]. Besides PCP, the pathogen may also cause other chronic severe lung infections, such as chronic obstructive pulmonary disease (COPD) and pose a risk to develop PCP-Immune Reconstitution Inflammatory Syndrome (PCP-IRIS) [12].

Diagnosis of PCP typically depends on screening of bronchoalveolar fluid (BAL) or induced sputum samples by use of conventional and/or fluorescent microscopy. To overcome the lack of sensitivity of microscopic analyses, especially in low burden non-HIV immunocompromised patients, the use of PCR-based analyses for diagnosis has increased [13,14]. Among others the mitochondrial ribosomal RNA large subunit (mtLSU) gene is used as a sensitive PCR target for PCP diagnosis [14]. The sensitivity of the mtLSU qPCR addresses the issue of the correlation between fungal tissue burden and clinical outcome, which is an important focus of research in order to discriminate between infection and colonization [15,16,17]. However, one has to take into account that patients with presumed colonization may even display chronic cough-like symptoms linked with *P. jirovecii* infections milder than PCP but nevertheless clinically relevant [18]. Although most studies have in common that higher *P. jirovecii* titers are related to PCP whereas lower titers are related to colonizations, none of these studies provide a standard universal cut-off [16,17].

The aim of the present study was to examine the reliability of mtLSU as a standard gene target in the quantitative diagnostic detection of *P. jirovecii*. For this reason, the mitochondrial single-copy gene COX1, which encodes the COX1 subunit of the cytochrome bc_1_ complex [19], as well as the nuclear single-copy genes KEX1 and DHPS were chosen as benchmarks. KEX1 encodes a kexin-like protease that likely causes the processing of proteins in the Golgi apparatus, and may also be involved in the proteolytic processing of MSG preceding transport to the cell surface [20,21]. Mitochondrial genes are subject to a nucleus-independent DNA replication process and thus behave differently regarding replication and inheritance compared to nuclear genes. Thus, ratios of nuclear to mitochondrial genes may give a hint of different life cycle stages of *P. jirovecii* leading to an improved diagnostic in the future.

## 2. Materials and Methods

### 2.1. Ethics Statement

This study was approved with a vote from the Ethical Committee of the Private University of Witten/Herdecke (vote no. 122/2016, 11 October 2016).

### 2.2. Samples

Bronchalveolar lavage fluids (BALF) were collected for routine diagnostic procedures to investigate the source of pneumonia and other respiratory tract infections from 2013–2017 at the Institute of Pathology (Merheim, Cologne, Germany). In our hospital BAL is a primary diagnosis making tool that in generally is performed in advance to therapy. From these samples we collected those with mtLSU positive PCR results obtained during our routine diagnostic procedures, for which we make use of the Botterel-qPCR, which is a confirmed and widely used detection protocol. We included two subgroups into the present study, a high mtLSU copy number group (>10^7^ mtLSU copies per mL) and a low copy number group (<10^7^ mtLSU copies per mL). The samples were retrospectively analyzed, double-blinded and were permitted to be used for retrospective analyses according to the Ethical Committee of the Private University of Witten/Herdecke (vote 122/2016), without the need for informed written consent.

Thirty-four *P. jirovecii* positive samples with more than 1 × 10^7^ *mtLSU* copies/mL were analyzed by additional PCRs targeting COX1, DHPS and KEX1. In case of 29 samples enough material was left for magnetic separation. In the second approach 50 samples with mtLSU titers <1 × 10^7^ copies/mL were randomly chosen and subjected to the novel CE-cleared IVD-labelled PneumoGenius^®^ assay (Pathonostics, Maastricht, The Netherlands).

### 2.3. Magnetic Labelling and Separation

The MACS^®^ Column Technology was used for the enrichment of MicroBead labelled *P. jirovecii* organisms. The magnetic separation was performed with LD Columns (Miltenyi Biotec, Bergisch Gladbach, Germany) according to the manufacturer’s instructions, with exception of applying a single wash with phosphate buffered saline (PBS) instead of a double wash. Stored BAL cell pellets were thawed, mixed and centrifuged at 300× *g* for 10 min at 4 °C. The pellet was resuspended in PBS (BioWhitaker^®^, Lonza, Verviers, Belgium). Then 330 µL cell suspension were labelled with 20 μL of the monoclonal mouse anti-*P. jirovecii* (*carinii*) IgM antibody 3F6 (A. Menari Diagnostics, Florence, Italy) and incubated overnight at 4 °C on a tube rotator. Then, 20 µL of anti-mouse IgM microbeads (Miltenyi Biotec, Bergisch Gladbach, Germany) were added to the suspension and incubated for a further 2 h at 4 °C under rotation. The purified collected flow-through was used for qPCR of two mitochondrial target genes (mtLSU and COX1) and two nuclear target genes (DHPS and KEX1).

### 2.4. DNA/RNA Extraction

DNA extractions were performed using the Maxwell^®^ Tissue LEV Total RNA Purification Kit, Custom (Promega Corporation, Madison, WI, USA) according to the manufacturer’s instructions.

### 2.5. Quantitative PCR

Quantitative PCR was performed to detect *P. jirovecii* using the Roche LightCycler 2.0 platform with two different protocols. Both were applied to filtered BALF DNA extracts as well as to MACS-purified DNA extracts.

For quantification serial dilutions of standard plasmids generated via ligation of the respective PCR-amplicons from reference material with the pCR4-TOPO vector (Invitrogen, Karlsruhe, Germany) were used. Plasmid DNA was purified with the Plasmid Maxi Kit (Qiagen, Hilden, Germany) according to manufacturer’s instructions and was subsequently sequenced. First, qPCR was performed to detect the mtLSU of *P. jirovecii* as formerly described by Botterel and co-workers [14]. Another qPCR amplified conserved regions of the nuclear encoded genes KEX1 (F: CTGGTTCGGAAGATGGAATAG, R: CAACTCAGCTACCCTAACTG) and DHPS (F: CGTTCTGAGGTTGCAGAACAA, R: GGAACTTTCAACTTGGCAACC) as well as the mitochondrial subunit COX1 gene (F: TCTCCTTCTGGTTGTTACCGC, R: ATCGACAGCACCTGAGGAATG). The PCR was set up in a final volume of 20 µL with 10 µL SYBR^®^ Green (QuantiTect^®^, Qiagen, Germany), 5 µL of extracted DNA or plasmid standard, 0.5 µL of the respective primers with 10 pmol each (Eurofins Genomics, Ebersberg, Germany) and 4 µL of PCR-grade water. The PCR program included a Taq activation step at 95 °C at 15 min. Amplification was performed for 55 cycles of denaturation (94 °C), annealing (55 °C) and extension (72 °C). The reference qPCR for quantification was the mtLSU-PCR from Botterel and colleagues, as this is a published, widely used protocol. The ratios between the different genes were calculated by a division of the copy numbers as previously described [22], with a sensitivity of 10 copies per mL.

### 2.6. Pneumogenius^®^ Assay

The PneumoGenius^®^ assay (Pathonostics, Maastricht, The Netherlands) was performed according to the manufacturer’s instructions. This multiplex real-time PCR assay detects *P. jirovecii* in respiratory tract samples by targeting the mtLSU and the DHPS domain of the FAS gene. Additionally, the PneumoGenius^®^ assay also determines the presence of two mutations that are associated with failure of treatment using sulfa-drugs. These mutations are located on codons 55 and 57 of the multi-domain FAS gene, encoding the enzyme dihydropteroate synthase (DHPS). The assay contains an internal assay control, is CE-marked and IVD labelled.

### 2.7. Statistical Analyses

It was assumed that the qPCR method by Botterel and coworkers provides the same results as a commercial IVD labelled test. To evaluate the reliability of the in house mtLSU qPCR compared to the PneumoGenius^®^ assay, a chi-square test with Yates correction was performed.

To test the hypothesis that different life cycle stages of *P. jirovecii* correlate with the ratios of COX-1, DHPS, and KEX-1 to mtLSU copy numbers, a simplified hierarchical clustering analysis was performed. The standardization was based on the mean values of the respective individual ratios including the standard deviation. The mean values were then used to calculate the Euclidean distances followed by hierarchical clustering of the results.

## 3. Results

At first 50 *P. jirovecii* positive tested samples with mtLSU titers <1 × 10^7^ copies/mL (Table 1) have been reanalyzed with the commercial non-quantitative PneumoGenius^®^ IVD assay. The PneumoGenius^®^ assay is not quantitative, but it showed a sensitivity of 96% compared to the in house mtLSU PCR regarding the presence of *P. jirovecii* in the analyzed BALs. The PneumoGenius^®^ assay that targets two different genes of the fungus delivered 42 positive *mtLSU* PCR results, but only 34 out of 50 cases were also positive for *DHPS*. In six of the *DHPS* positive cases the PneumoGenius^®^ assay was negative for mtLSU (Table 1).

Nevertheless, the PneumoGenius^®^ Assay could be considered positive for *P. jirovecii* in 48 of 50 cases. By two tailed chi square testing with Yates correction the chi squared equals 0.510 with 1 degree of freedom, while the two tailed *p*-value equals 0.4751. This means that the difference between the PneumoGenius^®^ and our in-house assay is not statistically significant.

By two tailed chi square testing with Yates correction the chi squared equals 75.041 with 1 degree of freedom, while the two tailed *p*-value is less than 0.0001, thus the difference between both assays is extremely statistically significant.

**Figure 1:**
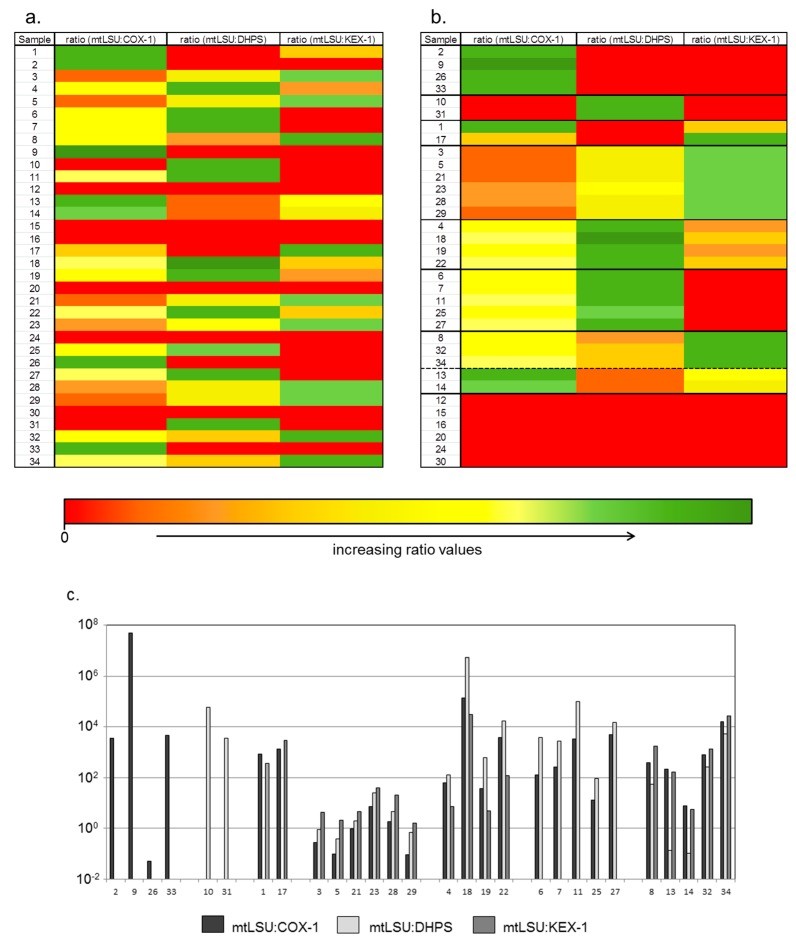
Copy number ratios of *COX1*, *KEX1* and *DHPS* vs. the diagnostic relevant *mtLSU* gene in 29 BALF samples with high titer of *mtLSU* gene copies (≥ 1 × 10^7^ *mtLSU* copies/mL). (**a**) Unsorted heatmap; (**b**) sorted heatmap; (**c**) According to their ratio pattern the samples were divided into seven clusters. In the clusters A and B only *COX1* or *DHPS* were detected besides *mtLSU*, whereas in the clusters C and F *DHPS* or *KEX1* are missing. In samples of the clusters D, E and G all target genes were detected, but each cluster displays a different ratio pattern.

These patterns were subject of a hierarchical clustering analyses based on Euclidian distance calculations that confirmed the patterns as distinct clusters. Thereby, each cluster has a minimum Euclidian distance >2 to at least one superior cluster (Figure 1).

In the clusters A and B only COX1 or DHPS were detected besides mtLSU, whereas in the clusters C and F DHPS or KEX1 are respectively missing. In samples of the clusters D, E and G all target genes were detected, but each cluster displays a specific ratio pattern. In pattern D, in all cases the ratio mtLSU:COX1 was lowest, followed by mtLSU:DHPS, which was higher, and the ration mtLSU:KEX-1 which was highest in this group. In cluster E, the ratio mtLSU:COX-1 is lower than mtLSU:DHPS but higher than mtLSU:KEX-1, while in cluster G mtLSU:COX-1 and mtLSU:KEX-1 are higher than mtLSU:DHPS.

Of the 34 samples, residual material was left from 29 samples for a subsequent MACS separation. Post MACS purification, there was an enrichment in the copy number observed in some cases, but in most cases the copy number decreased (Appendix A). Also, after separation of *P. jirovecii* particles the ratios of *COX1*, *KEX1* and *DHPS* to *mtLSU* range enormous between 9.01 × 10^4^ and 3.3 × 10^1^.

## 4. Discussion

Although there are conflicting reports and harmonized criteria for cut-off values are still missing [18,23,24,25], there is an increasing number of reports that the fungal load correlates with disease severity [26,27]. Among others the *mtLSU* gene is used as a PCR target to distinguish between colonization and infection [16,17]. As we previously observed serious differences in the ratio of mitochondrial to nuclear encoded genes of the fungus *Pneumocystis jirovecii* [28], a finding confirmed by recent publications [26,29], we have tried to address systematically the questions if a reliable quantification of *P. jirovecii* is possible with quantitative *mtLSU* PCR.

In conclusion, the *mtLSU* gene represents a sensitive qPCR target regarding the presence of *P. jirovecii* particles, which was also confirmed by a commercial assay with IVD labelling and CE clearance but does not display a perfect target for quantification. The quantification may have been influenced by anti-*Pneumocystis*-treatment in advance to the BAL sampling (e.g., echinocandins eliminate cysts but not trophozoites) and cannot fully excluded, although antifungal therapy is in general initiated after the laboratory diagnostics have been performed.

On the one hand one would expect that in a single sample almost similar copy numbers of *COX1* and *mtLSU* occur, as both genes are located as single copies on the mitochondrial genome [30], but in the described cohort that is not the case. On the other hand, for quantification the ratio of nuclear to mitochondrial genes should be approximately constant, since only single-copy nuclear genes are reliable molecular markers for the number of *Pneumocystis* particles, but the ratios in the analyzed patients vary within a range of several log steps. Explanations for these findings may be besides defective interfering mitochondrial DNA molecules indeed multicopies of mtLSU *in trans* supporting the assumption of Valero et al. that different mtDNA subspecies may exist dependent of the metabolic status [29]. The differences in the quantification between the MACS-purified and the native BAL samples can be explained by a simple loss of particles during the purification process or by a putative lack of binding capacity of the *P. jirovecii* specific antibodies used in the MACS separation. Although those antibodies are used in IVD assays and are deemed to bind all forms of the fungus in the entire life cycle, it cannot be excluded that some stages of the fungus are preferably bound and enriched.

Furthermore, we analyzed ratio patterns of *COX1*, *DHPS* and *KEX1* to *mtLSU* and identified different pattern clusters independent of the *mtLSU* load. This gives raise to the hypothesis that either several clinical strains with different compositions of mtDNA subspecies circulate, or that not only the metabolic status but also the stage within the fungal life cycle may influence the copy number ratios. In any case the applicability of *mtLSU* for quantitative analyses is ambiguous unless a correlation of all data collected to date can be experimentally proven. This in turn is hampered by the fact that neither a generally accepted cell culture nor a reliable animal model for human *Pneumocystis* strains exist. Although several PCR-assays have been developed and implemented, there are still a variety of gene targets to be explored to improve the outcomes of *P. jirovecii* infection. Regarding the actual findings one may speculate that the variety of ratio patterns, maybe representing different strains, may be one explanation for the latter mentioned difficulties in culturing this pathogen and the fact that not all *P. jirovecii* positive samples grow in cell cultures [28]. It also may be that culturing remains difficult as the pathogen does not survive the transfer from the patients’ body to the culture. In any case, further studies must include more patients on a multicenter basis and to evaluate if the above-mentioned clusters reflect different strains or subtypes. In addition, it will also be required to apply more sensitive techniques to also analyze the low mtLSU copy number isolates, which we not have included in our ratio analyses.

The variety of circulating strains is moreover underlined by the fact that the MACS purification resulted in altered overall detected copy numbers most likely due to specificity of the antibody used for separation. This implicates that the antibody either did not detect all strains in coinfections or that the antibody is more specific for special stages of the fungal life cycle. In this context, it has already been shown that differences in reactivity of monoclonal antibodies and polyclonal antisera reflect genetically distinct surface antigens of pneumocystis organisms in rat [31]. Although it was not our intention to contribute to the discussion of thresholds used to define or interpret laboratory findings to categorize patients into colonized or infected cohorts, one must realize that the mtLSU PCR is a highly sensitive diagnostic tool, but it may be not sufficient to calculate the effective fungal load of a sample.

Regarding the different biomarker profiles, the fact that no clinical data is included should not be interpreted as a study bias but is rather because we could not reach statistical significance, because we just had not enough *P. jirovecii* positive individuals in our cohort and for the respective clusters. It would be interesting to analyze their clinical significance in a future project with an appropriate number of patient cases and to evaluate whether the clusters represent strains with different pathogenicity, different life cycle stages or metabolic state.

Despite all speculations the recent study shows that more questions arise the more detailed this pathogen is investigated, and that besides basic research also applied research and harmonization of methods must be performed to establish reliable quantitative diagnostics of *P. jirovecii.*

## Figures and Tables

**Table 1:** Results of the PneumoGenius^®^ assay and the mtLSU PCR according to Botterell [14]. “+” positive tested, “⌀” negative tested.

	mtLSU	PneumoGenius^®^			mtLSU	PneumoGenius^®^	
		mtLSU	DHPS			mtLSU	DHPS
1	2.52 × 10^5^	+	+	26	6.48 × 10^3^	+	⌀
2	7.55 × 10^4^	+	+	27	2.45 × 10^6^	+	+
3	1.36 × 10^6^	+	⌀	28	1.05 × 10^6^	+	+
4	1.38 × 10^6^	+	⌀	29	3.12 × 10^6^	+	+
5	4.41 × 10^4^	⌀	+	30	4.15 × 10^6^	+	+
6	3.22 × 10^5^	+	⌀	31	7.84 × 10^6^	+	+
7	4.02 × 10^4^	+	+	32	7.50 × 10^4^	⌀	⌀
8	2.20 × 10^2^	+	⌀	33	1.95 × 10^5^	+	+
9	1.21	+	⌀	34	2.21 × 10^6^	+	+
10	9.26 × 10^6^	+	+	35	2.22 × 10^6^	+	+
11	7.63 × 10^6^	+	+	36	3.36 × 10^4^	+	+
12	1.12 × 10^6^	+	⌀	37	8.76 × 10^6^	+	+
13	4.52 × 10^6^	+	+	38	2.87 × 10^6^	+	+
14	6.92 × 10^3^	+	+	39	1.08 × 10^6^	+	+
15	1.02 × 10^4^	+	+	40	1.00 × 10^6^	⌀	+
16	5.71 × 10^5^	+	⌀	41	6.73 × 10^6^	+	⌀
17	9.77 × 10^6^	+	+	42	1.65 × 10^6^	⌀	+
18	4.33 × 10^5^	+	+	43	2.78 × 10^5^	+	+
19	1.52 × 10^6^	+	+	44	2.60 × 10^6^	+	+
20	1.43 × 10^5^	+	+	45	1.55 × 10^5^	⌀	+
21	3.19 × 10^3^	⌀	⌀	46	4.29 × 10^6^	+	⌀
22	4.60 × 10^3^	+	+	47	1.12 × 10^3^	+	+
23	1.58 × 10^4^	⌀	+	48	1.42 × 10^4^	⌀	+
24	3.77 × 10^3^	+	+	49	8.35 × 10^3^	+	⌀
25	9.27 × 10^3^	+	⌀	50	6.92 × 10^3^	+	⌀

## Appendix A

**Table A1:** Results of qPCRs for nuclear and mitochondrial gene copy number. Results of qPCRs for nuclear and mitochondrial gene copy numbers in 34 BALF samples with high titer of *mtLSU* gene copies (≥1 × 10^7^ *mtLSU* copies/mL) before and after MACS purification of *P. jirovecii* particles. In case of samples 13, 18, 28, 29 and 31 no material was left for magnetic separation. After MACS purification of *P. jirovecii* particles three samples showed a positive fungal proof and two samples showed a positive bacterial proof in the respective endpoint PCRs. n/a not applicable.

Sample No.	*mtLSU* (Copies/mL)		*COX1* (Copies/mL)		*KEX1* (Copies/ mL)		*DHPS* (Copies/mL)	
	Pre-Separation	Post-Separation	Pre-Purification	Post-Purification	Pre-Purification	Post-Purification	Pre-Purification	Post-Purification
1	4.33 × 10^8^	negative	5.22 × 10^5^	negative	1.21 × 10^6^	negative	negative	negative
2	2.16 × 10^7^	negative	5.93 × 10^3^	negative	negative	negative	negative	negative
3	9.02 × 10^8^	1.30 × 10^7^	3.33 × 10^9^	3.14 × 10^8^	2.08 × 10^8^	2.19 × 10^9^	9.97 × 10^8^	7.04 × 10^7^
4	9.52 × 10^7^	3.30 × 10^4^	1.53 × 10^6^	1.82 × 10^5^	1.30 × 10^7^	negative	7.41 × 10^5^	negative
5	7.99 × 10^9^	2.20 × 10^5^	8.12 × 10^10^	5.18 × 10^7^	3.77 × 10^9^	1.40 × 10^8^	2.04 × 10^10^	9.44 × 10^6^
6	1.99 × 10^9^	8.20 × 10^4^	1.56 × 10^7^	1.14 × 10^6^	n/a	negative	5.21 × 10^5^	2.73 × 10^1^
7	3.50 × 10^7^	negative	1.36 × 10^5^	negative	n/a	negative	1.30 × 10^4^	4.22 × 10^3^
8	2.62 × 10^7^	negative	6.56 × 10^4^	9.51 × 10^4^	1.47 × 10^4^	negative	4.71 × 10^5^	negative
9	2.33 × 10^9^	6.20 × 10^4^	4.79 × 10^1^	negative	negative	negative	negative	negative
10	1.47 × 10^8^	negative	negative	negative	negative	negative	2.54 × 10^3^	negative
11	2.11 × 10^9^	1.70 × 10^11^	6.26 × 10^5^	1.89 × 10^5^	negative	negative	2.16 × 10^4^	negative
12	5.79 × 10^8^	negative	negative	negative	negative	negative	negative	negative
13	3.32 × 10^12^	n/a	1.49 × 10^10^	n/a	2.05 × 10^10^	n/a	2.52 × 10^13^	n/a
14	1.03 × 10^10^	1.30 × 10^7^	1.37 × 10^9^	1.75 × 10^9^	1.84 × 10^9^	8.41 × 10^9^	1.00 × 10^11^	4.04 × 10^9^
15	1.13 × 10^8^	negative	negative	negative	negative	5.71 × 10^7^	negative	negative
16	4.33 × 10^9^	negative	negative	negative	negative	negative	negative	negative
17	5.99 × 10^8^	3.60 × 10^11^	4.52 × 10^5^	2.90 × 10^4^	2.06 × 10^5^	negative	negative	negative
18	1.61 × 10^14^	n/a	1.14 × 10^9^	n/a	5.04 × 10^9^	n/a	3.04 × 10^7^	n/a
19	1.59 × 10^10^	1.40 × 10^6^	4.20 × 10^8^	2.22 × 10^8^	3.24 × 10^9^	9.40 × 10^8^	2.59 × 10^7^	2.25 × 10^8^
20	2.70 × 10^7^	2.10 × 10^2^	negative	negative	negative	negative	negative	negative
21	2.98 × 10^11^	8.60 × 10^6^	3.05 × 10^11^	1.03 × 10^9^	6.45 × 10^10^	9.55 × 10^9^	1.53 × 10^11^	7.11 × 10^9^
22	1.05 × 10^12^	1.90 × 10^6^	2.74 × 10^8^	1.96 × 10^8^	8.51 × 10^9^	9.07 × 10^8^	6.29 × 10^7^	3.14 × 10^8^
23	1.01 × 10^11^	3.50 × 10^7^	1.44 × 10^10^	4.82 × 10^8^	2.52 × 10^9^	5.89 × 10^8^	3.91 × 10^9^	3.47 × 10^6^
24	9.92 × 10^8^	4.00 × 10^14^	negative	negative	negative	negative	negative	1.21 × 10^4^
25	2.11 × 10^7^	3.70 × 10^5^	1.67 × 10^6^	8.37 × 10^4^	negative	negative	2.34 × 10^5^	negative
26	1.09 × 10^10^	6.50 × 10^8^	2.17 × 10^11^	1.05 × 10^10^	n/a	5.64 × 10^10^	n/a	2.25 × 10^9^
27	1.65 × 10^8^	negative	3.24 × 10^4^	negative	negative	negative	1.11 × 10^4^	9.39 × 10^3^
28	1.07 × 10^8^	n/a	5.92 × 10^7^	n/a	5.23 × 106	n/a	2.39 × 10^7^	n/a
29	1.04 × 10^9^	n/a	1.17 × 10^10^	n/a	6.56 × 10^8^	n/a	1.55 × 10^9^	n/a
30	2.88 × 10^7^	5.70 × 10^3^	negative	negative	negative	negative	negative	4.66 × 10^3^
31	1.79 × 10^7^	n/a	negative	n/a	negative	n/a	4.83 × 10^3^	n/a
32	9.90 × 10^8^	negative	1.21 × 10^6^	negative	7.41 × 10^5^	negative	3.79 × 10^6^	negative
33	2.80 × 10^8^	negative	6.20 × 10^4^	negative	negative	negative	negative	2.08 × 10^4^
34	1.98 × 10^10^	6.20 × 10^5^	1.21 × 10^6^	5.00 × 10^6^	7.41 × 10^5^	2.66 × 10^7^	3.79 × 10^6^	2.85 × 10^6^
